# Graphene-Doped Poly (Methyl-Methacrylate) (Pmma) Implants: A Micro-CT and Histomorphometrical Study in Rabbits

**DOI:** 10.3390/ijms22031441

**Published:** 2021-02-01

**Authors:** Antonio Scarano, Tiziana Orsini, Fabio Di Carlo, Luca Valbonetti, Felice Lorusso

**Affiliations:** 1 Department of Innovative Technologies in Medicine & Dentistry, University of Chieti-Pescara, Via dei Vestini 31, 66100 Chieti, Italy; dicarlofabio61@gmail.com (F.D.C.); felice.lorusso@unich.it (F.L.); 2 Department of Medical, Oral and Biotechnological Sciences, University of Chieti-Pescara, Via dei Vestini 31, 66100 Chieti, Italy; 3 CNR—National Research Council, Institute of Biochemistry and Cell Biology, 00015 Monterotondo Scalo (RM), Italy; tiziana.orsini@cnr.it; 4 DVM, Unit of Basic and Applied Biosciences, Faculty of Veterinary Medicine, University of Teramo, 64100 Teramo, Italy; lvalbonetti@unite.it

**Keywords:** graphene-doped PMMA, PMMA, graphene, osseointegration, micro-CT

## Abstract

Background—the graphene-doping procedure represents a useful procedure to improve the mechanical, physical and biological response of several Polymethyl methacrylate (PMMA)-derived polymers and biomaterials for dental applications. The aim of this study was to evaluate osseointegration of Graphene doped Poly(methyl methacrylate) (GD-PMMA) compared with PMMA as potential materials for dental implant devices. Methods—eighteen adult New Zealand white male rabbits with a mean weight of approx. 3000 g were used in this research. A total of eighteen implants of 3.5 mm diameter and 11 mm length in GD-PMMA and eighteen implants in PMMA were used. The implants were placed into the articular femoral knee joint. The animals were sacrificed after 15, 30 and 60 days and the specimens were evaluated by µCT and histomorphometry. Results—microscopically, all 36 implants, 18 in PMMA and 18 in DG-PMMA were well-integrated into the bone. The implants were in contact with cortical bone along the upper threads, while the lower threads were in contact with either newly formed bone or with marrow spaces. The histomorphometry and µCT evaluation showed that the GP-PMMA and PMMA implants were well osseointegrated and the bone was in direct contact with large portions of the implant surfaces, including the space in the medullary canal. Conclusions—in conclusion, the results suggest that GD-PMMA titanium surfaces enhance osseointegration in rabbit femurs. This encourages further research to obtain GD-PMMA with a greater radiopacity. Also, further in vitro and vivo animal studies are necessary to evaluate a potential clinical usage for dental implant applications.

## 1. Introduction

The recent trends of the literature in the field of implant dentistry report an increase of implant titanium-supported rehabilitation for the treatment of partial or complete edentulism [1,2] with high success rate after a five-year period [3,4]. However, titanium implants have limited osseointegration and osteoinductive properties, particularly in cases of insufficient or poor bone conditions. Sandblasting and/or acid etching techniques have been used for increasing the biological responses of cells around titanium. Another strategy is to load the titanium with hydroxyapatite (HA), Ca [5] and other coatings. Also, calcium, magnesium, strontium and zinc have been injected into the titanium for enhancing osseointegration through cell adhesion and proliferation of osteogenic cells [6,7,8]. These treatments have high costs that limit their applications. Still, titanium has a low tendency for corrosion but electrical, mechanical and chemical factors have an effect on the peri-implant microbiota and the health of the peri-implant tissues [9]. Despite the overall promising outcomes there is need for innovative materials with high corrosion resistance, lack of metal [10] and with anti-plaque activity to avoid bone loss and peri-implantitis [11].

The dynamic of the scientific research on biomaterials in oral and maxillofacial surgery is oriented towards cost-effective new generation materials with ideal aesthetic, physical and mechanical properties, modulus of elasticity, thermal and electrical features, and ideal porosity to substitute the damaged hard tissue defects, such as human dentine and bone [12].

These new generation biomaterials should have biocompatibility properties and induce the adhesion and differentiation of the osteoprogenitor cells and generate osseointegration of the interface [13,14]. Polymethyl methacrylate (PMMA)-based polymers represent a group of resins commonly used in implant dentistry for several clinical uses, such as temporary crowns, sealants, restorative bonding, composites, cements, dentures, orthodontics devices, splinting materials and maxillofacial prosthetics [15].

Moreover, PMMA-derived materials have also been applied in orthopaedic surgery as bone cement and in craniofacial implant restoration for the treatment of bone defects [16,17,18].

In this way, PMMA polymers have been proposed as dental implant materials, with excellent aesthetic and clinical outcomes [19].

The autopolymerising resins based on polymethyl methacrylate (PMMA) are the materials most used in dentistry, due to low resistance to impact and a low transversal and flexion resistance, derived from the formation and the spread of cracks when they are put through mechanical stress [20].

It is well known that the mechanical and chemical features of polymer-based materials can be oriented by doping the polymer with a suitable material, in order to produce a significant increase in their features for clinical applications [21]. PMMA is an interesting material, first studied in 1901 by Dr. Otto Röhm, that was used in different fields, such as in aeronautical engineering and, after many years, was introduced into the medical field for cranial reconstruction and in dentistry for its high mechanical resistance.

Graphene has raised wide interest thanks to its thermal, mechanical, electrical and other properties. The addition of oxygen atoms bound with the carbon scaffold create a new compound called graphene oxide (GO). G is hydrophobic in nature while GO is hydrophilic, that is, easily dispersible in water. It is characterized by a carbon-based material with mono-atomic layer thickness, considered as the first two-dimensional (2D) crystal. This structure confers graphene an exceptionally high mechanical stiffness [22] and an extraordinary high thermal [23] and electrical conductivity [24]. It is set to go beyond all the other allotropes in utility for life and material sciences due to its several intrinsic properties. For this reason, it is used as a coating of materials and biomaterials that usually lack these characteristics, so that they can be used in a wide variety of applications in different fields with excellent performances. Graphene coating has been proposed as a coating of bone scaffolds and biomaterials in order to improve cell adhesion, proliferation and differentiation characteristics [25]. The particles of porcine coated by graphene oxide (GO) are able to improve the mechanical bone healing property [26].

Graphene is the ideal candidate for improving the performance of autopolymerising acrylic resins for dental use, not only due to its high traction resistance, coefficient of thermal expansion, high capacity for absorption and lubrication, flexibility and high surface area, but also for its high resistance ratio [21].

The aim of this in vivo investigation was to evaluate the bone formation around PMMA and GD-PMMA implants in rabbit keen-joints. The tested null hypothesis was that the GD-PMMA biocomplex used in a dental implant could induce a same osseointegration as the PMMA implant.

## 2. Results

### 2.1. Scanning Electron Microscopy

**Figure 1:**
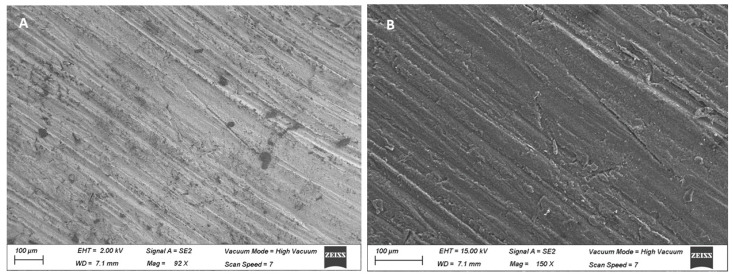
(**A**) Polymethyl methacrylate (PMMA) disk. (**B**) Graphene doped (GD)-PMMA. Streaks imparted by the milling were present at low magnification.

In both groups, PMMA and GD-PMMA, the topography of the disks showed the typical streaks imparted by the milling (Figure 1).

### 2.2. Atomic Force Microscopy (AFM)

In both cases the topography of the disks showed the typical streaks imparted by the milling. For 10 × 10 micrometer areas, the Sdr roughness parameter (that indicates the % increase of actual surface area with respect to the geometrical one) was 18 ± 7 for the PMMA sample and 17 ± 3 for the GD-PMMA, without significant difference.

PMMA Implant—the roughness parameters evaluated gave mean values of Ra: 0.72 ± 0.1 µm and Rq: 0.9 ± 0.3 µm.

GD-PMMA Implant—the roughness parameters evaluated gave mean values of Ra: 0.73 ± 0.1 µm and mean Rq: 0.9 ± 0.5 µm.

This result suggests that effect on the bone is not dictated by differences in surface topography, rather it genuinely reflects the contribution of the different surface chemistries to new peri-implant bone formation.

### 2.3. Histological Evaluation

**Figure 2:**
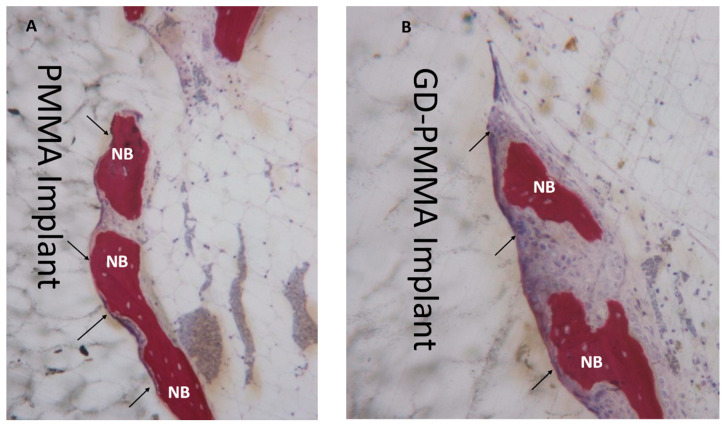
(**A**) PMMA implant. New bone (NB) in direct contact with implant (arrows) is present. Acid fuchsin-toluidine blue 100×. (**B**) GD-PMMA implant. Osteoblast (arrows) and new bone (NB) in direct contact with the implant. Acid fuchsin-toluidine blue 100×.

**Figure 3:**
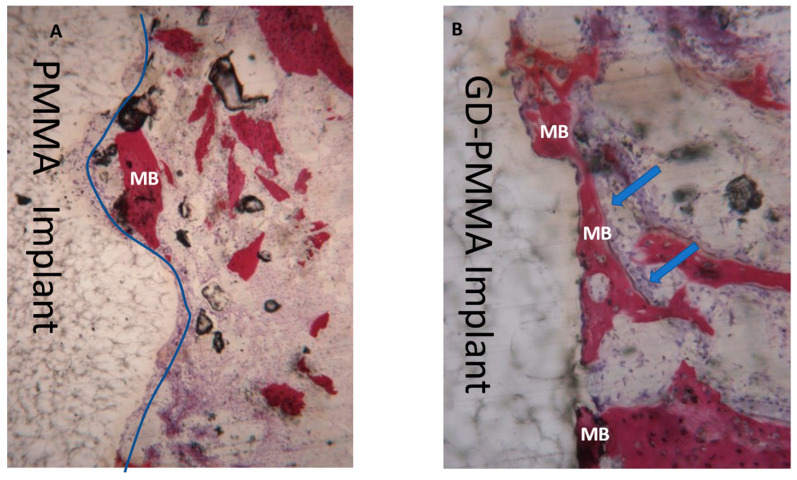
(**A**) PMMA implant. Mature bone (MB) in direct contact with the implant (blue line) is present. Acid fuchsin-toluidine blue 100×. (**B**) GD-PMMA implant. Mature bone (MB) in direct contact with the implant and osteoblast activity (arrows). No degradation layers or cracks and detachment of fragments are observed in either implant. Acid fuchsin-toluidine blue 100×.

**Figure 4:**
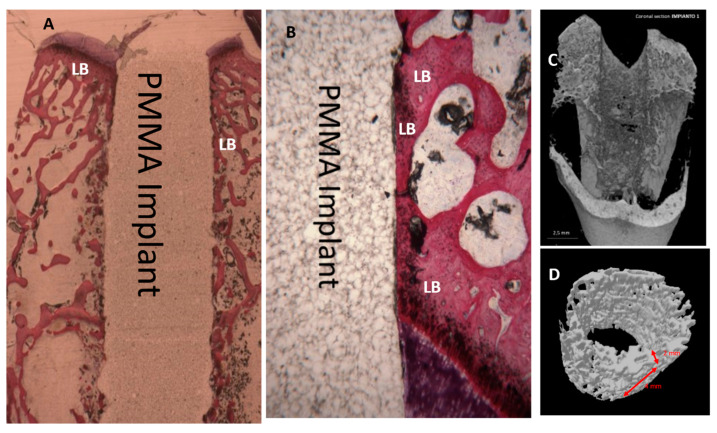
PMMA implant. (**A**) Lamellar bone (LB) in direct contact with the implant is present. Acid fuchsin-toluidine blue 12×. (**B**) Mature bone (LB) in direct contact with the implant, no osteoblast activity is present. No degradation layers or cracks nor detachment of fragments are observed. Acid fuchsin-toluidine blue 100×. (**C**,**D**) Micro-CT shows a mineralized bone with small medullary space.

**Figure 5:**
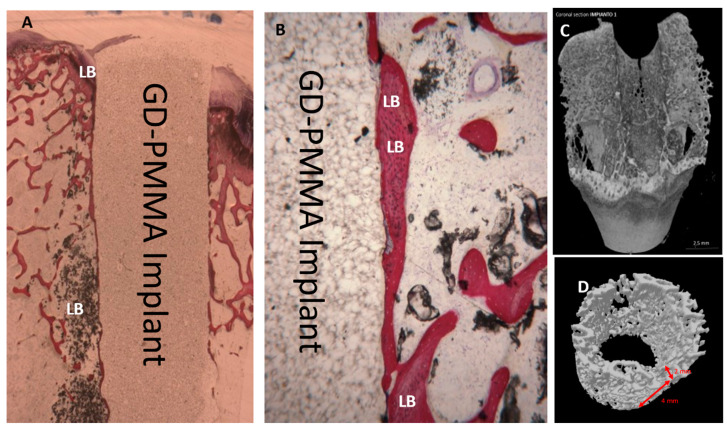
A GD-PMMA implant. (**A**) Lamellar bone (LB) in direct contact with the implant. Acid fuchsin-toluidine blue 12× (**B**) Mature bone (LM) in close contact with the implant, no osteoblasts or macrophages are observed. Acid fuchsin-toluidine blue 50× (**C**,**D**) Micro-C shows mineralized bone in direct contact with the implant.

**Figure 6:**
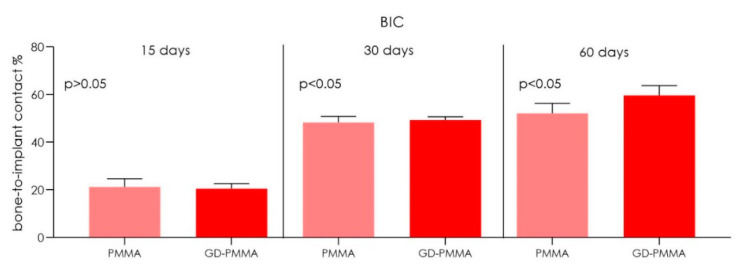
Chart of histomorphometric bone-to-implant contact percentage (BIC%) between the PMMA implants group and GD-PMMA implants group. A significant difference was detected between PMMA and GD-PMMA groups at 30 days and 60 days (Kruskal Wallis followed by the Bunn’s test).

**Figure 7:**
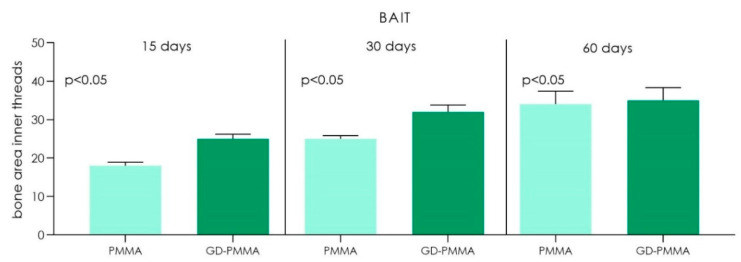
Chart of volume of bone area inner threads (BAIT) between the PMMA implants group and GD-PMMA implants group. A significant difference was detected between PMMA and GD-PMMA groups at 15, 30 and 60 days (Kruskal Wallis followed by the Bunn’s test).

**Figure 8:**
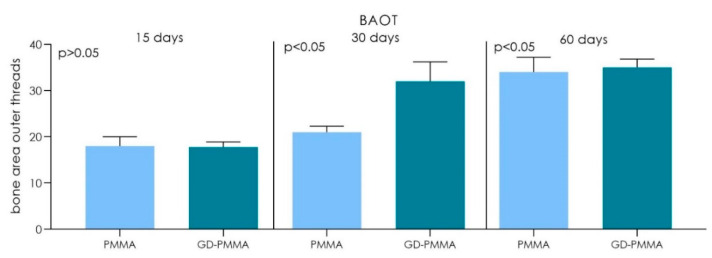
Chart of volume of bone area outer threads (BAOT) between the PMMA implants group and GD-PMMA implants group. A significant difference was detected between PMMA and GD-PMMA groups at 30 and 60 days (Kruskal Wallis followed by the Bunn’s test).

Microscopically, all 36 implants, 18 in PMMA and 18 in DG-PMMA, were well-integrated into bone and appear white due to the light of the optical microscope. Implants were in contact with cortical bone along the upper threads, while the lower threads were in contact with either newly formed bone or with marrow spaces. Fibrous tissue was absent between bone and implant surfaces in all the implants of the two groups (Figure 2, Figure 3, Figure 4, Figure 5, Figure 6, Figure 7 and Figure 8, Table 1). No foreign body reaction or infiltrate pathological cells were observed. Representative histological pictures of the samples at day 15, 30 and 60 are shown in Figure 2, Figure 3, Figure 4, Figure 5, Figure 6, Figure 7 and Figure 8 and Table 1.

#### 2.3.1. 15 Days

After 15 days in both implants there was an intense osteoblastic activity and woven bone. Native bone had been remodelled, and a few osteoclasts were observed. A larger osteon, not yet organized into secondary osteons, was observed in the bone. No degradation layers or cracks and detachment of fragments were observed at each experimental time. It can be observed that new bone was in contact with the implant only in a few areas. New bone showed a trabecular morphology, with large osteocytes lacunae (Figure 2).

The histomorphometry analysis showed:

PMMA implant. The mean BIC percentage was 21.22 ± 3.5%, bone area inner threads (BAIT) was 18 ± 0.9% and bone area outer threads (BAOT) was 18 ± 0.2%.

GD-PMMA implant. The mean BIC percentage was 20.5 ± 2.1%, bone area inner threads (BAIT) was 25 ± 1.2% and bone area outer threads (BAOT) was 17.8 ± 1.1%.

#### 2.3.2. 30 Days

After 30 days, both implants showed a mature bone, and only a few areas of osteoblast activity. The newly formed bone in contact with the dental implant and cement lines formed with the original bone were visible, appearing already mineralized with a similar density to the surrounding bone. In many areas new mature bone was observed in contact with the implant (Figure 3).

The histomorphometry analysis showed:

PMMA implant. The mean BIC percentage was 48.2 ± 2.6%, bone area inner threads (BAIT) was 25 ± 0.8% and bone area outer threads (BAOT) was 21 ± 1.3%.

GD-PMMA implant. The mean BIC percentage was 49.3 ± 1.4%, bone area inner threads (BAIT) was 32 ± 1.8% and bone area outer threads (BAOT) was 32 ± 4.2% (Table 2 and Table 3).

#### 2.3.3. 60 Days

After 60 days, only lamellar bone was observed around both implants. No bone resorption or osteolysis was observed. In many areas lamellar bone in contact with the implant could be observed (Figure 4 and Figure 5).

The histomorphometry analysis showed:

PMMA implant. The mean BIC percentage was 55.1 ± 4.2%, bone area inner threads (BAIT) was 34 ± 3.4% and bone area outer threads (BAOT) was 34 ± 3.2% (Table 2 and Table 3).

GD-PMMA implant. The mean BIC percentage was 59.6 ± 4.1%, bone area inner threads (BAIT) was 35 ± 3.3% and bone area outer threads (BAOT) was 35 ± 1.8%.

### 2.4. Micro-CT Evaluation

**Figure 9:**
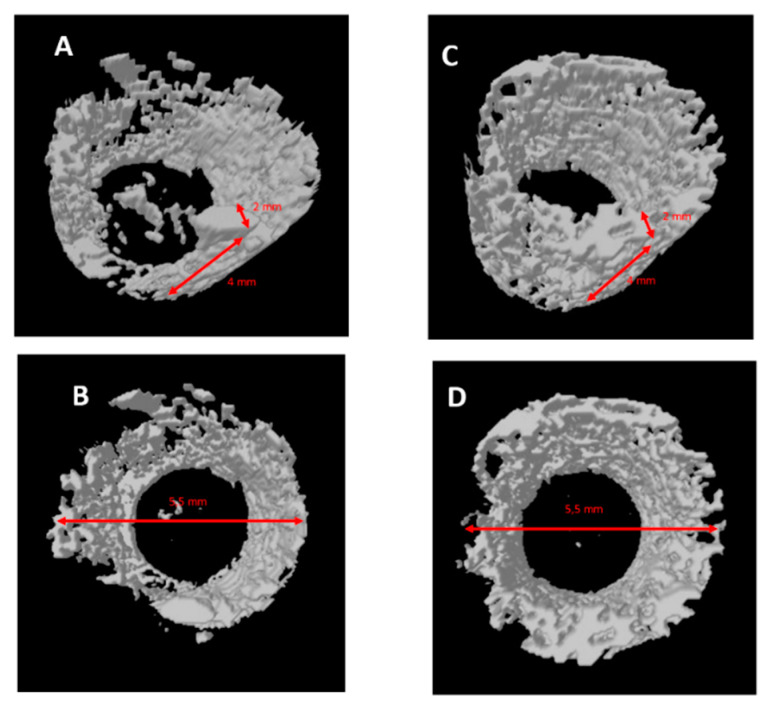
(**A**,**B**) PMMA implant. Micro-CT shows a mineralized bone with large medullary space. (**C**,**D**) GD-PMMA implant. Micro-C shows mineralized bone in direct contact with the implant.

Reconstructed μCT images allowed the visualization of the bone in 3D, with high spatial resolution and high-density contrast. No artefacts from the implant samples were detectable after 15 days in either group. No degradation layers nor cracks nor detachment of fragments were observed at each experimental time. The volume of interest (VOI) (3D), 2 mm thick and 4 mm (85 slides) high, was generated for each implant, obtaining a hollow cylinder on which the automatic three-dimensional calculation showed new bone in both implants in intimate contact with the implant surface, no soft tissues were detected at any time. No signs of bone resorption, osteolysis and/or inflammation were observed on either surface. After 15 days it could be observed that new bone was in contact with the implant only in a few areas. New bone showed a trabecular morphology, with large osteocytes lacunae (Figure 9).

**Figure 10:**
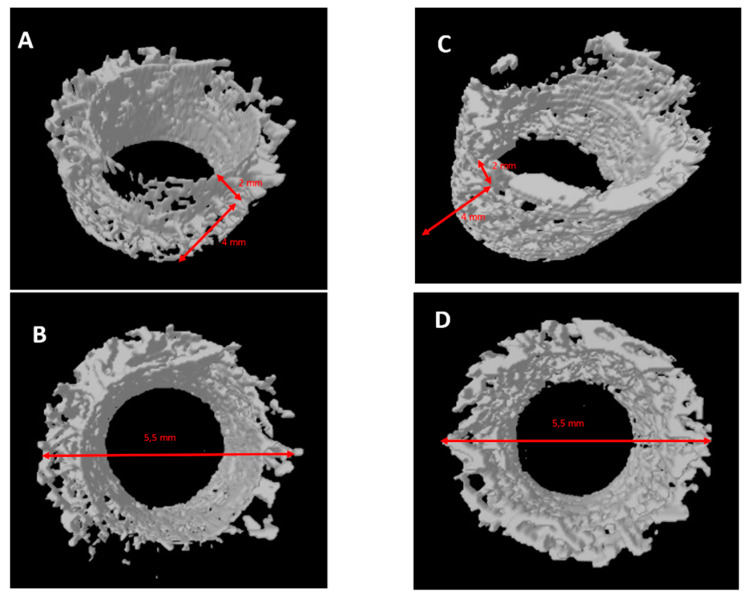
(**A**,**B**) PMMA implant. Micro-CT shows a mineralized bone with small medullary space. (**C**,**D**) GD-PMMA implant. Micro-C shows mineralized bone in direct contact with the implant.

After 30 days in both groups, it could be observed that new bone was in contact with the implant only in a few areas. New bone showed a trabecular morphology, with small osteocyte lacunae (Figure 10).

**Figure 11:**
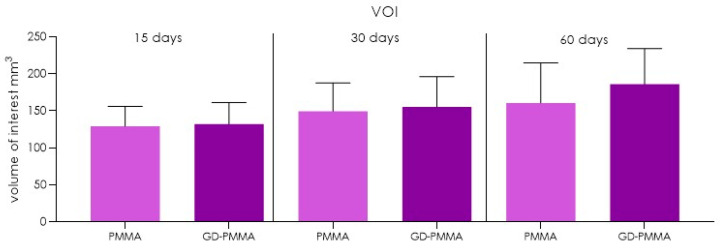
Chart of volume of interest (VOI) between the PMMA implants group and GD-PMMA implants group. A significant difference was detected between PMMA and GD-PMMA groups at 60 days (one-way ANOVA-Tukey’s post hoc test).

After 60 days in both groups, it could be observed that lamellar bone was in contact with the implant in many areas (Figure 4 and Figure 5). It had a matured morphology and had the same density as the native bone. The GP-PMMA and PMMA implants were well osseointegrated and bone was in direct contact with large portions of the implant surfaces, including the space in the medullary canal. Representative pictures of the samples at day 15, 30 and 60 time points are shown in Figure 11. The VOI (3D), 2 mm thick and 4 mm showed (Table 4):

After 15 days

PMMA: Total VOI volume, TV, 1.2902 × 10^11^, µm^3^

GD-PMMA: Total VOI volume, TV, 1.3202 × 10^11^, µm^3^

After 30 days

PMMA: Total VOI volume, TV, 1.4926 × 10^11^, µm^3^

GD-PMMA: Total VOI volume, TV, 1.5502 × 10^11^, µm^3^

After 60 days

PMMA: Total VOI volume, TV, 1.6051 × 10^11^, µm^3^

GD-PMMA: Total VOI volume, TV, 1.8577 × 10^11^, µm^3^

## 3. Discussion

The outcome of this study showed a good osseointegration in both groups with statistical difference in bone-to-implant contact percentage (BIC%) and micro-CT variables bone area inner threads (BAIT), bone area outer threads (BAOT) and volume of interest (VOI) between the PMMA implant and GD-PMMA implant groups. Both implants had a similar roughness without significant difference. These results suggest that the effect on the bone healing is not dictated by differences in surface topography, rather, it genuinely reflects the contribution of the different surface chemistries to peri-implant new bone formation. Histomorphometry results also confirmed, through µCT, this evaluation, which showed an increase of the total bone at 2 and 4 mm from the implant.

Graphene-doped PMMA is a material that can be milled following a CAD-Cam procedure in order to customize the shape of the device, that potentially suggests, for a clinical application, the possibility to adapt the implant fixture to the anatomy and the defects of the alveolar bone ridge. Moreover, the CAD-Cam technique is related to drastically low amounts of methacrylate monomer [27]. Polymethylmethacrylate (PMMA) is used in percutaneous vertebroplasty for filling bone marrow cavities or gaps between bone and implants, given its injectability, high mechanical properties and biocompatibility [28,29].

PMMA was used as a membrane for treating the critical size defect in rabbit calvaria and the authors showed that it is a potential material for a guided tissue regeneration membrane [30].

The outcomes of this study indicate that GD-PMMA implants could provide a similar osseointegration to sandblasted and acid etched titanium [14]. It is important to point out that the implant used in this research was only milled by following CAD-Cam procedure, without treatment of the surface. However, physical and chemical surface treatments were used to increase the quality and quantity of bone in contact with the titanium implant.

Probably, the various methods used for implant surface treatments to enhance the performance of titanium implants could be used for further increasing osseointegration of DG-PMMA. Another strategy could be to bind bioactive molecules already used on titanium such as Collagen type I [8]. In the present study we have used the PMMA doped with graphene to increase the differentiation of pre-osteoblast cells and to enhance the protein adsorption, hydrophilicity, osseointegration capacity and osteogenesis in vivo [31]. Titanium coated with graphene oxide (GO) can be loaded onto the outermost surface with bioactive molecules such us BMP-2 for better and new osteogenesis on a titanium fixture when implanted into a mouse calvaria [32]. Graphene was used as coating in a titanium implant, enhancing calcium deposition and osteoinduction, and as a stem cell recruitment agent, improving quality and quantity of bone around and in contact with the implant [33]. Graphene coating also improved the anti-corrosion properties of titanium [34,35,36].

Acid fuchsin and toluidine blue staining of bone samples showed different results between woven bone and old or mature bone. In both experimental groups, there was newly formed bone in contact with implant materials and after 30 days a mature bone was observed. In conclusion, DG-PMMA has a good osseointegration property. It induces no adverse tissue reaction and effectively supports selective bone regeneration.

The null hypothesis was rejected because the GD-PMMA biocomplex used as a dental implant induces a better osseointegration when compared to the PMMA implant especially in the BAIT, BOAT and VOI.

However, the GD-PMMA biocomplex induces a similar osseointegration to titanium when compared with a previous study conducted in a rabbit model [14,37].

The roughness surface of both implants used in the present study is comparable to machined titanium. This indicating that probably the bone response could be further enhanced after sandblasting and acid-etching implant surface treatment, treatments already used on titanium implants to improve the mechanical fixation between the bone and implant after placement.

It should be noted that this material had one limitation in dental implantology for its radiolucency. However, this study encourages further studies in order to obtain implants in DG-PMMA with high radiopacity and with treatment of the surface such as sandblasting and acid-etching.

## 4. Materials and Methods

### 4.1. Scanning Electron Microscopy

Five PMMA disks and five GD-PMMA disks were analysed using scanning electron microscopy (Carl Zeiss, Oberkochen, Germany). All samples were 5 mm in diameter and 3 mm in height.

### 4.2. Atomic Force Microscopy (AFM)

Five PMMA and five GD-PMMA disks were used for the evaluation of the surface roughness by AFM. All the disks were 4 mm in diameter and 2 mm in height. Measurements were performed using a NX10 Park AFM instrument (Park System, Suwon, Korea), equipped with 20-bit closed-loop XY and Z flexure scanners and a non-contact cantilever PPP-NCHR 5M. This device implements a True Non-Contact^TM^ mode, allowing minimization of the tip-sample interaction, resulting in tip preservation, negligible disk nanotopography modification and reduction of artefacts. The Flatten command was used to eliminates unwanted features from scan lines (noise, bow and tilt). The filter was used prior to image analysis commands to eliminate image low frequency noise, which appear as horizontal shifts or stripes in the image. On each sample, three different areas were analyzed at a scan rate of 0.1 Hz. Surface topography parameters were acquired using the software, Atomic Force Microscope NX10 Park (Park Systems Suwon Korea).

### 4.3. In Vivo Experiment

Eighteen adult New Zealand white male rabbits with a mean weight of approx. 3000 g were used in this study. The protocol was approved by the local Ethics Committee of the University of Chieti-Pescara, Chieti, Italy and by the Italian Ministry of Health n°525/219-PR. The study was performed in accordance with the relevant guidelines and regulations of Italian law animal research and followed the international guidelines for animal treatment and conformed to the ARRIVE guidelines [38,39]. A total of eighteen implants of 3.5 diameter and 11 mm length in GD-PMMA were used. The implant is provided by a cylindrical macro-geometry. The first coronal part is characterized by a length of 0.8 mm, while the thread morphology is characterized by a pitch of 1 mm with a conical apical portion. Sterilization of the implants was done by exposure to dry heat at 121 °C for 1 h. After sterilization, the implants were immediately placed. The rabbits were obtained from a commercial source (CHARLES RIVER LABORATORIES, Lecco Italy), judged clinically healthy by a veterinarian and housed at the Vivarium of the University of Chieti-Pescara, Italy. The animals were held under veterinary supervision in standardized rabbit cages (only one rabbit per cage), maintained in a laboratory environment with regulated temperature (21–24.5 °C), humidity (42–57%), and 12-hourly dark and light cycles All the animals had ad libitum access to a regular rabbit chow and water.

**Figure 12:**
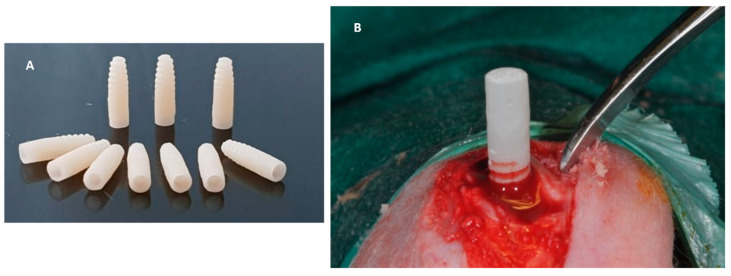
PMMA and GD-PMMA before and during placement in the bone knee-joint.

The implants were placed into the articular femoral knee joint. All the animals were moved from the rabbit vivarium to the surgical operating room. Each animal before the surgical procedure was anesthetized with intramuscular injections of diazepam (1.5 mg/kg b.wt.) and fluanizone (0.7 mg/kg b.wt.), and local anaesthesia was given using 1 mL of 2% articaine/adrenalin solution (CURADEN HEALTHCARE S.R.L, Saronno, Italy). The skin of the leg was shaved and disinfected with 2% chlorhexidine gluconate antiseptic solution (CURADEN HEALTHCARE S.R.L, Saronno, Italy) to achieve asepsis of the skin. A skin cut with a periosteal flap was used to expose the articular surface. The implant bed was prepared using a conventional dental handpiece with a physio-dispenser (Vario-Surgery NSK, Tochigi, Japan) with drills cooled by saline irrigation. Each rabbit received two implants, one in each knee-joint—PMMA in the left knee and GD-PMMA in the right knee-joint (Figure 12). A total of eighteen implants of 3.5 mm diameter and 11 mm length in GD-PMMA and eighteen implants in PMMA were used. No animal died during the post-operative time and they could walk without visible signs of discomfort, pain, infection or wound dehiscence. The animals were sacrificed after 15, 30 and 60 days, by placing them in a CO_2_ saturated chamber. A total of 36 implants were retrieved.

### 4.4. Specimen Processing

The rabbit femurs were removed and were dissected from the soft tissue. The knee-joints were washed previously in saline solution and after 10 min were immediately put in 4% paraformaldehyde and 0.1% glutaraldehyde in 0.15 M cacodylate buffer al 4 C and pH 7.4 at room temperature for 1 week, for fixation of tissues and for processing to achieve specimen histology. The ground sections with the SCAN 1 Automated System (Pescara, Italy) were used for achieving the section containing the implant. The samples were dehydrated in ascending concentrations of ethyl alcohol from 60% to 100% rinses and embedded in a hydrophilic acrylic resin of high viscosity (LR White Resin London Resin Company Ltd., Hampshire, UK). This resin had been polymerized with the activator and the block and was sectioned, along the longitudinal axis, with a diamond disc at approx. 140 µm, and ground down to approx. 40 µm with a specially designed grinding machine to obtain 2 sections. Slides with 40 µ thick histological sections were stained using toluidine blue and acid fuchsin, as previously described [40]. The histological section was then observed in transmitted light by a Nikon microscope ECLIPSE (Nikon, Tokyo, Japan). The aspects of the newly formed and mature bone could be classified in consequence of the histological color of the tissues (light red = old matrix, dark red = new matrix) and their quantity was expressed in percentage (mean ± SD). The microscope was connected to a video camera with high-resolution, a high-definition monitor and a computer workstation (Notebook Hp). A dedicated software package for image capturing was used for morphometric evaluation by digital image-analysis (NIS-Elements AR 3.0 software, Nikon, Minato, Japan). The bone-to-implant contact (BIC) percentages, bone area inner threads (BAIT) and bone area outside threads (BAOT) were calculated as described in a previous study [8].

### 4.5. Statistical Evaluation

The statistical analysis of the study data was performed using the software package Graphpad 8 (Prism, San Diego, CA, USA). The data descriptive statistic was expressed as means, standard deviations and 95% confidence interval (CI). The study variables of bone-to-implant contact (BIC) percentages, bone area inner threads (BAIT) and bone area outside threads (BAOT) were analyzed the non-parametric Kruskal Wallis followed by the Bunn’s test to perform the intergroups comparisons. The level of significance was considered as *p* < 0.05.

### 4.6. Micro-CT Evaluation

Tomographic analysis of samples was performed using the high-resolution 3D imaging system Skyscan 1172G (Bruker MicroCT, Kontich, Belgium). The acquisition of volumes was obtained with 0.5 mm Al filter, image pixel/size of 21.96 µm, camera binning 4 × 4, source voltage of 70 kV, source current of 141 µA, exposure time of 500 ms. The reconstructed tomographic volumes of the acquired scans were generated by a built-in NRecon Skyscan reconstruction software (Version: 1.6.6.0; Skyscan Bruker), while volume rendering and virtual sectioning views as well as imaging analysis were developed using 3D Visualization Softwares CTvox v. 2.5, DataViewer v. 1.4.4 (Skyscan Bruker), CT-Analyser software version 1.13 (Skyscan Bruker) and ImageJ.

A VOI (3D), 2 mm thick and 4 mm (85 slides) high, was generated for each implant, obtaining a hollow cylinder on which the automatic three-dimensional calculation was launched.

## 5. Conclusions

In conclusion, the results suggest that the GD-PMMA implant enhances osseointegration in rabbit femurs which encourages further research to obtain a GD-MMA with a greater radiopacity. Further in vitro and in vivo animal studies are necessary to evaluate a potential clinical usage for dental implant applications.

## Figures and Tables

**Table 1:** Summary of histomorphometric bone-to-implant contact percentage (BIC%) between the PMMA implants group and GD-PMMA implants group (Kruskal Wallis followed by the Bunn’s test).

BIC	15 Days				30 Days				60 Days			
	PMMA		GD-PMMA		PMMA		GD-PMMA		PMMA		GD-PMMA	
Mean (SD)	21.22	(3.5)	20.5	(2.1)	48.2	(2.6)	49.3	(1.4)	52.1	(4.2)	59.6	(4.1)
95% CI	(17.52–24.92)		(18.26–22.74)		(45.51–50.89)		(47.8–50.8)		(50.65–59.55)		(55.25–63.95)	
*p* value	*p* > 0.05				*p* < 0.05				*p* < 0.05			

**Table 2:** Summary of histomorphometric micro-CT variables bone area inner threads (BAIT) between the PMMA implants group and GD-PMMA implants group (Kruskal Wallis followed by the Bunn’s test).

BAIT	15 Days				30 Days				60 Days			
	PMMA		GD-PMMA		PMMA		GD-PMMA		PMMA		GD-PMMA	
Mean (SD)	18	(0.9)	25	(1.2)	25	(0.8)	32	(1.8)	34	(3.4)	35	(3.3)
95% CI	(17.07–18.93)		(23.78–26.22)		(24.18–25.82)		(30.16–33.84)		(30.47–37.53)		(21.57–28.43)	
*p* value	*p* < 0.05				*p* < 0.05				*p* < 0.05			

**Table 3:** Summary of histomorphometric micro-CT bone area outer threads (BAOT) between the PMMA implants group and GD-PMMA implants (Kruskal Wallis followed by the Bunn’s test).

BAOT	15 Days				30 Days				60 Days			
	PMMA		GD-PMMA		PMMA		GD-PMMA		PMMA		GD-PMMA	
Mean (SD)	18	(2)	17.8	(1.1)	21	(1.3)	32	(4.2)	34	(3.2)	35	(1.8)
95% CI	(17.78–18.22)		(16.69–18.91)		(19.64–22.36)		(27.56–36.44)		(30.47–37.53)		(21.57–28.43)	
*p* value	*p* > 0.05				*p* < 0.05				*p* < 0.05			

**Table 4:** Summary of histomorphometric micro-CT volume of interest (VOI) between the PMMA implants group and GD-PMMA implants group (Kruskal Wallis followed by the Bunn’s test).

VOI	15 Days				30 Days				60 Days			
	PMMA		GD-PMMA		PMMA		GD-PMMA		PMMA		GD-PMMA	
Mean (SD)	129	(27.0)	132	(29.0)	149.2	(38.1)	155	(41.2)	160.5	(54.1)	185.7	(48.1)
95% CI	(100.3–157.7)		(101.4–162.6)		(109.2–189.2)		(111.7–198.3)		(103.7–217.3)		(135.3–236.1)	
*p* value	*p* > 0.05				*p* > 0.05				*p* < 0.05			

## Data Availability

All experimental data to support the findings of this study are available contacting the corresponding author upon request. The authors have annotated the entire data building process and empirical techniques presented in the paper. The data underlying this article are not freely available to protect their confidentiality.
